# Prevalence and Molecular Characterization of *Cryptosporidium* Species in Patients With Hematologic Malignancies in Eastern Iran

**DOI:** 10.1155/ijm/3566454

**Published:** 2026-05-25

**Authors:** Rahmat Solgi, Seyed Mohammad Mousavi, Sadaf Saeedi, Nima Firouzeh, Amir Tavakoli Kareshk, Tahereh Davoodi

**Affiliations:** ^1^ Infectious Diseases Research Center, Birjand University of Medical Sciences, Birjand, Iran, bums.ac.ir; ^2^ Research Center for Hydatid Disease in Iran, Kerman University of Medical Sciences, Kerman, Iran, kmu.ac.ir; ^3^ Vector-Borne Diseases Research Center, North Khorasan University of Medical Sciences, Bojnurd, Iran, nkums.ac.ir; ^4^ Department of Medical Sciences, Islamic Azad University, Kazerun Branch, Kaserun, Iran, ctb.iau.ir

**Keywords:** *Cryptosporidium parvum*, genotyping, hematologic malignancy, Iran

## Abstract

**Introduction:**

*Cryptosporidium* is a leading protozoan cause of diarrheal disease globally, posing a significant threat to immunocompromised individuals, including patients with hematologic malignancies. This study was aimed at finding out how common *Cryptosporidium* is, what species and genotypes are present, and what risk factors are linked to patients with leukemia and lymphoma in Birjand, South Khorasan. This region has not had this information available before.

**Methods:**

In this cross‐sectional study conducted in 2023, stool samples were collected from 55 patients with confirmed hematologic cancers at Iranmehr Hospital, Birjand. Samples were examined using microscopy (modified Ziehl–Neelsen stain), and all were subsequently analyzed using a nested PCR assay targeting the *gp60* gene for species and subtype identification. Demographic and risk factor data were collected and analyzed for association with infection using Fisher′s exact test.

**Results:**

The overall prevalence of cryptosporidiosis was 3.6% (two out of 55 cases). One case was found in a patient with leukemia (one out of 34; 2.9%), and another in a patient with lymphoma (one out of 21; 4.8%). The difference in prevalence between these groups was not statistically significant. Molecular analysis identified all isolates as *Cryptosporidium parvum*. Sequencing revealed two zoonotic subtypes: IIaA in the lymphoma patient and IIdA in the leukemia patient. A statistically significant association was found between infection and contact with animals (*p* = 0.004) and having a high‐risk occupation (*p* = 0.03).

**Conclusion:**

As the first report in our study area, we identified a 3.6% prevalence of cryptosporidiosis among patients with hematologic malignancies in Birjand, with zoonotic *C. parvum* subtypes as the sole etiological agents. The results emphasize the significant role of zoonotic transmission in this population and indicate a critical need for tailored preventive counseling to mitigate exposure risks associated with potential animal reservoirs for these vulnerable patients.

## 1. Introduction

*Cryptosporidium*, an intracellular protozoan parasite, is recognized worldwide as a significant cause of diarrheal disease and associated mortality [1]. The parasite′s oocysts, known for their resilience, show high resistance to standard environmental disinfectants such as chlorine, posing a significant challenge to the control of cryptosporidiosis [2]. Transmission to humans occurs primarily via the fecal–oral route, facilitated through various pathways, including direct person‐to‐person contact, zoonotic spread from infected animals, and ingestion of contaminated food or water, often leading to large‐scale outbreaks [3]. Notably, animals, particularly young livestock, play a crucial role as asymptomatic reservoirs, shedding oocysts into the environment and contaminating water sources and food supplies, highlighting the need for comprehensive control strategies [4]. Upon ingestion, *Cryptosporidium* oocysts excyst in the gastrointestinal tract, liberating sporozoites that penetrate the epithelial cells of the small intestine. This generally interferes with normal absorptive mechanisms and undermines mucosal defenses, resulting in cryptosporidiosis. Clinically, this condition predominantly presents as either acute or chronic gastroenteritis [5]. The severity of the disease is significantly influenced by host‐related factors, with age and immune status emerging as the primary determinants of disease outcomes [6]. While infection in immunocompetent individuals typically results in a self‐limiting diarrheal illness, it can become severe, persistent, and life‐threatening in immunocompromised populations [7]. High‐risk groups include young children, the elderly, and individuals with immune deficiencies [8]. Patients with hematologic malignancies, such as leukemia and lymphoma, represent a particularly vulnerable immunocompromised cohort [9]. Their susceptibility to opportunistic infections is multifactorial, stemming from the inherent immunosuppressive nature of the malignancy itself, which disrupts the normal function of immune cells, and from the profound myelosuppressive and lymphotoxic effects of treatments like chemotherapy, radiotherapy, and steroid use [10]. In these individuals, cryptosporidiosis may present as a persistent and debilitating diarrheal condition, resulting in malabsorption, significant weight loss, and spread to other organs such as the biliary and respiratory systems, which greatly increases morbidity and mortality [11]. Research examining *Cryptosporidium* in this patient population has shown a broad range of prevalence, varying from approximately 2.7% to more than 20%. This variability can be attributed to factors such as geographic differences, the diagnostic techniques employed, and the unique characteristics of the patients studied [9, 12]. The advent of molecular epidemiology has revolutionized our understanding of *Cryptosporidium*. To date, over 40 species and 120 genotypes have been identified, with at least 20 species known to infect humans [2]. The two most common species in humans are *Cryptosporidium hominis*, which is largely restricted to humans and transmitted via an anthroponotic cycle, and *Cryptosporidium parvum*, a zoonotic species with a broad host range including cattle and other livestock [13]. Distinguishing between species and genotypes is crucial for identifying infection sources, understanding transmission dynamics, and implementing effective public health controls. For instance, the predominance of *C. hominis* often points to human‐to‐human transmission or waterborne outbreaks from human sewage contamination, whereas the presence of *C. parvum* suggests zoonotic or environmental transmission from animal reservoirs [14]. The geographic distribution of *Cryptosporidium* species varies. Studies have shown that *C. hominis* is often the dominant species in developing countries, while both *C. hominis* and *C. parvum* are common in developed nations [13]. This distribution is also evident among immunocompromised patients. For example, a study in Thailand on HIV‐positive patients found *C. hominis* to be the most frequent species, followed by *Cryptosporidium meleagridis* and *Cryptosporidium canis* [14]. Molecular studies within Iran and neighboring regions have documented both anthroponotic and zoonotic patterns. Some studies from Iran reported predominance of *C. hominis*, others *C. parvum* [15, 16], while international reports highlight IIa and IId subtype families as frequent findings in human infections with zoonotic links [14, 17]. This variability highlights the need for localized molecular surveillance to better understand transmission dynamics in specific provinces. South Khorasan Province—with mixed urban and agricultural/rural communities and frequent human–livestock interactions—has lacked specific molecular data on *Cryptosporidium* in populations with hematologic malignancy. This designed project is aimed at filling this knowledge gap by integrating microscopy and nested PCR, which focuses on the gp60 gene, along with sequencing for subtyping. This approach is used to analyze the distribution of species and subtypes in patients with leukemia and lymphoma at Iranmehr Hospital in Birjand during 2023. Gaining insights into the distribution of species and gp60 subtypes is crucial for enhancing clinical practices, implementing effective infection control in oncology environments, and addressing public health initiatives related to water safety and the transmission of infections from animals to humans. The study is aimed at (i) estimating the prevalence of *Cryptosporidium* infection in hematologic cancer patients in this setting, (ii) identifying species and gp60 subtypes, and (iii) exploring associations with demographic and exposure variables.

## 2. Methods

### 2.1. Ethical Considerations

The study protocol was reviewed and approved by the Ethics Committee of Birjand University of Medical Sciences (Ethical Code: IR.BUMS.REC.1402.219). The objectives and procedures of the study were explained to all potential participants, and written informed consent was obtained before enrolment. Confidentiality of patient data was maintained throughout the study. Prior to initiation of the study, institutional permission to recruit patients and collect stool samples was obtained from the administration of Iranmehr Hospital, Birjand. The objectives and procedures of the study were explained to all potential participants, and written informed consent was obtained before enrollment. All samples and associated clinical data were anonymized before analysis, and confidentiality of patient information was maintained throughout the study.

### 2.2. Study Design and Population

The present descriptive, cross‐sectional study was conducted between spring and winter of 2023. The study population comprised patients diagnosed with hematologic malignancies (leukemia and lymphoma) who were referred to Iranmehr Hospital in Birjand, the capital of South Khorasan Province, Iran. A total of 55 patients were enrolled in the study based on convenience sampling over the 1‐year period.

### 2.3. Inclusion and Exclusion Criteria

To be eligible for the study, participants needed to be diagnosed with leukemia or lymphoma by a certified oncologist, be aged between 18 and 70, and give their full informed consent to participate. Individuals who opted out of the study, provided inadequate stool samples for analysis, or had other existing chronic health issues were excluded from participation.

### 2.4. Data and Sample Collection

Demographic and risk factor data were collected from each participant using a standardized checklist. Information gathered included age, gender, place of residence (urban/rural), educational level, occupation, contact with domestic or farm animals, and source of drinking water. A single stool sample was collected from each of the 55 patients in a sterile, wide‐mouthed container.

### 2.5. Laboratory Procedures

#### 2.5.1. Microscopic Examination

**Figure 1:**
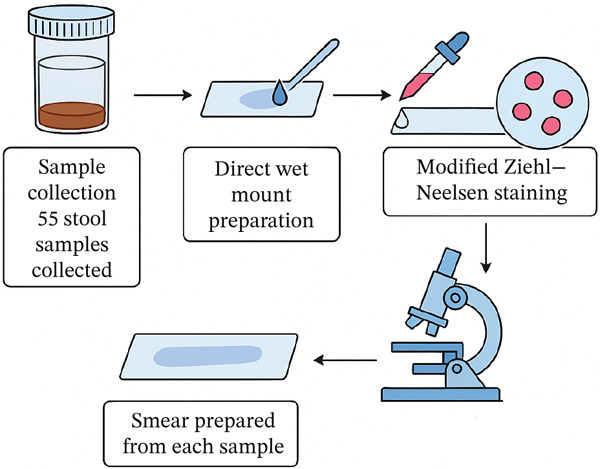
The schematic description regarding the modified Ziehl–Neelsen staining protocol.

All 55 stool samples were initially examined for the presence of *Cryptosporidium* oocysts. A direct wet mount preparation was performed, and a smear from each sample was stained using the modified Ziehl–Neelsen (acid‐fast) technique. Slides were examined under a light microscope at 1000x magnification to identify the characteristic pink‐to‐red spherical oocysts (schematic protocol was depicted in Figure 1).

### 2.6. Sample Preservation and DNA Extraction

Stool samples that were positive by microscopy, as well as all other samples for molecular analysis, were preserved at 4°C. An aliquot was also preserved in 70% ethanol for long‐term storage. Genomic DNA was extracted from approximately 200 mg of each stool sample using a commercial DNA extraction kit (Favorgen Biotech Corp., Taiwan) according to the manufacturer′s protocol. The extracted DNA was stored at −20°C until use.

### 2.7. Molecular Analysis: Nested PCR and Genotyping

A nested PCR assay targeting a fragment of the 60 kDa glycoprotein (*gp60*) gene was used for the definitive identification and subtyping of *Cryptosporidium*. The first PCR round amplified an ~854 bp fragment using the forward primer Gp60‐eF (5 ^′^‐ATA GTC TCC GCT GTA TTC‐3 ^′^) and the reverse primer Gp60‐eR (5 ^′^‐GCA GAG GAA CCA GCA TC‐3 ^′^). The second, nested PCR round used the product of the first round as a template to amplify an internal ~467 bp fragment, using the forward primer Gp60‐iF (5 ^′^‐TCC GCT GTA TTC TCA GCC‐3 ^′^) and the reverse primer Gp60‐iR (5 ^′^‐GAG ATATAT CTT GGT GCG‐3 ^′^). The final PCR products were visualized by electrophoresis on a 1.5% agarose gel stained with a safe DNA stain. Products of the correct size from positive samples were purified and sent to a commercial facility for bidirectional sequencing. The resulting DNA sequences were analyzed using BLASTn against the GenBank database to determine the *Cryptosporidium* species and subtype family. These primers have been reported to amplify the gp60 locus in these two major human pathogenic species and, in some studies, in a subset of other *Cryptosporidium* species.

### 2.8. Statistical Analysis

All collected data were entered and analyzed using SPSS software, Version 21.0 (IBM Corp., Armonk, NY, United States). Descriptive statistics, including frequencies and percentages, were used to summarize demographic and clinical data. Fisher′s exact test was used for analytical comparisons to assess the association between *Cryptosporidium* infection and categorical variables (e.g., gender, animal contact, and cancer type). A *p* value of less than 0.05 was considered statistically significant.

## 3. Results

### 3.1. Study Population and Demographic Characteristics

A total of 55 stool samples were collected from patients with hematologic malignancies (lymphoma and leukemia) referred to Iranmehr Hospital in Birjand, Iran, between spring and winter 2023. The demographic characteristics of the participants are summarized in Table 1. Of the 55 patients, 35 (63.8%) were male and 20 (36.2%) were female. The mean age was not calculated, but the highest frequency was observed in the 40–60 years age group (40.0%). All participants (100%) reported using purified drinking water as their primary source. Contact with domestic animals (e.g., dogs, cats, cows, and sheep) was reported by four individuals (7.2%). Education levels varied, with the highest prevalence among those with an illiterate or primary education (45.4%). Most participants resided in urban areas (48; 87.3%), and 45 (81.8%) had occupations unrelated to intestinal parasitic infections. Statistical analysis revealed no significant associations between cryptosporidiosis infection and most demographic variables, including sex, age, education level, residence, or drinking water source (all *p* > 0.05). In exploratory analyses using Fisher′s exact test, infection was more frequent among participants reporting contact with animals (two of four; 50.0%) and among those in high‐risk occupations (*p* = 0.004 and *p* = 0.03, respectively). However, these findings are based on only two infected cases and very small cell counts and therefore should be interpreted with caution.

**Table 1:** Association between demographic characteristics, risk factors, and *Cryptosporidium* infection in patients with hematologic malignancies (*n* = 55).

Variable	Category	Total *n* (%)	Infected *n* (%)	*p* value
Gender	Male	35 (63.6)	2 (5.7)	0.5
	Female	20 (36.4)	0 (0.0)	

Residence	Urban	48 (87.3)	1 (2.1)	0.2
	Rural	7 (12.7)	1 (14.3)	

Age group (years)	< 40	19 (34.5)	0 (0.0)	1.0
	41–60	22 (40.0)	2 (9.1)	
	> 60	14 (25.5)	0 (0.0)	

Contact with animals	Yes	4 (7.3)	2 (50.0)	0.004
	No	51 (92.7)	0 (0.0)	

Occupation	Related risk^a^	10 (18.2)	2 (20.0)	0.03
	Unrelated risk	45 (81.8)	0 (0.0)	

Cancer type	Leukemia	34 (61.8)	1 (2.9)	0.7
	Lymphoma	21 (38.2)	1 (4.8)	

^a^Related risk occupations include farming and animal husbandry.

### 3.2. Prevalence of *Cryptosporidium* Infection

The overall prevalence of *Cryptosporidium* infection, as determined by microscopic examination (direct method and Ziehl–Neelsen staining), was 3.6% (2/55 samples). Infection rates differed by malignancy type: 1.6% (1/55) in leukemia patients and 2.5% (1/55) in lymphoma patients, with no significant difference between groups (*p* > 0.05; Table 1).

### 3.3. Microscopic Findings

Microscopic evaluation identified several intestinal protozoa (Table 2). Among pathogenic protozoa, *Giardia lamblia* was the most common (3.6%; 2/55), followed by *Microsporidia* (1.8%; 1/55) and *Cryptosporidium* (3.6%; 2/55). Nonpathogenic protozoa included *Blastocystis* (5.4%; 3/55), *Entamoeba coli* (7.2%; 4/55), *Chilomastix mesnili* (3.6%; 2/55), and *Endolimax nana* (1.8%; 1/55).

**Table 2:** Prevalence of intestinal protozoa identified by microscopy (*N* = 55).

Protozoan	Pathogenic/nonpathogenic	Frequency (*n*)	Percentage (%)
*Giardia lamblia*	Pathogenic	2	3.6
*Microsporidia*	Pathogenic	1	1.8
*Cryptosporidium*	Pathogenic	2	3.6
*Blastocystis*	Nonpathogenic	3	5.4
*Entamoeba coli*	Nonpathogenic	4	7.2
*Chilomastix mesnili*	Nonpathogenic	2	3.6
*Endolimax nana*	Nonpathogenic	1	1.8

### 3.4. Molecular Findings

**Figure 2:**
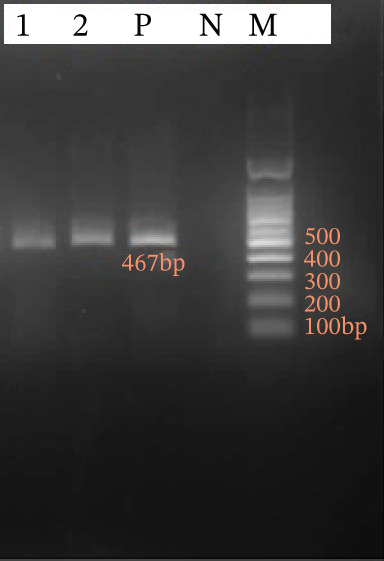
Agarose gel electrophoresis (1.5%) of nested PCR products for the gp60 gene. Lanes 1–2: patient samples (positive for *Cryptosporidium*); Lane P: positive control; Lane N: negative control; Lane M: 100 bp marker.

Of the two microscopically positive *Cryptosporidium* samples, both were confirmed positive by nested PCR targeting the gp60 gene, producing bands of approximately 854 bp (first round) and 467 bp (second round) (Figure 2). In addition, 60 microscopy‐negative samples (30 harboring other intestinal protozoa and 30 microscopy‐negative controls) were tested by nested PCR, and all were negative for *Cryptosporidium*. Subtype analysis revealed one sample as IIaA (50%) and one as IIdA (50%). Genotype distribution by malignancy type showed one IIaA in a lymphoma patient and one IIdA in a leukemia patient, with no significant difference (Fisher′s exact test, *p* = 1.000; Table 3).

**Table 3:** Comparison of *Cryptosporidium* genotypes by malignancy type.

Cancer type	IIaA (*n*, %)	IIdA (*n*, %)	*p* value
Leukemia (*n* = 34)	1 (2.9)	0 (0.0)	1.000
Lymphoma (*n* = 21)	0 (0.0)	1 (4.8)	1.000

## 4. Discussion

This study detected *Cryptosporidium* infection in two of 55 (3.6%) patients with hematologic malignancies attending Iranmehr Hospital, Birjand, during 2023. Molecular subtyping of gp60 showed that both isolates belonged to *C. parvum* Subtypes IIaA and IIdA (one sample each). Contact with animals and occupation categorized as infection‐related were associated with infection, whereas sex, age group, residence category, and reported drinking water source were not significantly associated. Sequencing phylogeny placed isolates in clusters consistent with zoonotic lineages. These findings suggest a relatively low prevalence, but with evidence consistent with zoonotic transmission in this cohort. The overall prevalence of 3.6% in our survey is consistent with findings from other studies on immunocompromised patients in Iran. For instance, Pestechian et al. [16] reported a similar prevalence of 3.4% among cancer patients in Isfahan, and Faridi et al. [15] found a 3.6% prevalence in patients with hematologic malignancies as part of a larger study on immunocompromised individuals in central Iran. The prevalence observed in our study is within the 3.6%–5.4% range reported for various population groups in Iran, suggesting that while these cancer patients are highly susceptible, the infection rate reflects the general endemicity in the region. However, this rate is lower than the 10% reported in a study on hematologic malignancy patients in Poland [18] and significantly lower than rates reported in some studies in developing nations, which can approach 40% in certain risk groups [19]. These variations are probably due to differences in geographic regions, sanitation practices, local farming methods, and the diagnostic techniques used. A key finding of this study is the exclusive detection of *C. parvum*. This contrasts with many global reports, particularly from other developing regions and studies on HIV‐positive individuals, where the anthroponotic species *C. hominis* is often dominant [14, 20]. For example, studies in Thailand and Lebanon identified *C. hominis* as the predominant species, pointing to human‐to‐human transmission cycles. The absence of *C. hominis* in our cohort strongly suggests that the primary transmission dynamic in this specific geographic and demographic setting is zoonotic rather than anthroponotic. This conclusion is further reinforced by our risk factor analysis, which identified a highly significant association between infection and animal contact (*p* = 0.004), as both infected patients reported such contact. This direct link between the epidemiological data and our molecular results strengthens the evidence for a zoonotic transmission pathway in this population. The identification of the specific *C. parvum* subtypes provides further insight into the sources of infection. The subtypes found, IIaA and IIdA, are two of the most globally prevalent zoonotic subtypes responsible for human cryptosporidiosis. The IIa family is frequently associated with cattle, particularly calves, while the IId family is also commonly found in cattle and other small ruminants like sheep and goats [21]. Our findings are consistent with studies from other regions where livestock are implicated as major reservoirs. For example, *C. parvum* IIa and IId subtypes have been reported as dominant zoonotic agents in studies from Algeria [17], Scotland [22], and other parts of Iran [23, 24]. The presence of these specific subtypes in patients with leukemia and lymphoma in Birjand, a province where animal husbandry is common, reinforces the conclusion that livestock serve as the main reservoir for human infections. Infection likely occurs through direct contact with infected animals or indirectly through the contamination of the environment, food, or water with oocysts from animal feces. From a clinical and public health perspective, these findings are of significant importance. They highlight that patients undergoing treatment for hematologic malignancies in South Khorasan are at risk from an environmental and agricultural source. This implies that preventive strategies must extend beyond standard hospital infection control and focus on patient education. Oncologists and healthcare providers should counsel these highly susceptible patients to avoid direct contact with livestock, especially young or diarrheic animals, and to practice meticulous personal hygiene after any potential environmental exposure. Although our study did not find a significant difference in prevalence between leukemia (2.9%) and lymphoma (4.8%) patients, this is likely due to the small number of positive cases. The overarching risk is the state of severe immunosuppression common to both conditions, which obliterates the immune system′s ability to control this opportunistic pathogen. This study has several strengths. It is the first to use molecular tools to investigate *Cryptosporidium* in this specific high‐risk group in the region, and it successfully utilized the robust *gp60* gene for subtyping, which provides clear data on transmission pathways. However, the study is not without limitations. The primary limitation is the small sample size (*N* = 55) and the use of convenience sampling, which may affect the generalizability of our findings and the precision of the prevalence estimate. The low number of positive cases (*n* = 2) also constrains the statistical power of the risk factor analysis and prevents any definitive conclusions about associations between specific genotypes and clinical outcomes or cancer types. Future research should aim to overcome these limitations. Larger, multicenter studies across the province are needed to establish a more accurate prevalence rate and to analyze risk factors with greater statistical power. Furthermore, a “One Health” approach, involving the concurrent screening of local livestock (cattle, sheep, and goats) and environmental water sources for *Cryptosporidium*, would be invaluable. Such studies could confirm the specific animal reservoirs for the IIaA and IIdA subtypes found in humans and definitively map the transmission pathways in the region, leading to more targeted and effective public health interventions.

## 5. Conclusion

This study provides the first molecular evidence of *Cryptosporidium* infection and gp60 subtypes in patients with hematologic malignancies in Birjand, South Khorasan. We detected *C. parvum* infections in 3.6% (2/55) of patients, with zoonotic IIaA and IIdA subtypes, and observed that infected individuals reported occupational and animal exposures consistent with livestock‐associated transmission. Occupational animal exposure correlated with infection. Despite the low prevalence, these findings highlight the necessity for increased clinical vigilance, systematic diagnostic testing in symptomatic oncology patients, and a One Health approach that integrates human, animal, and environmental sampling to mitigate transmission risks.

## 6. Limitations

The most important limitation of this study is the small sample size (*N* = 55), combined with the use of convenience sampling at a single referral hospital. Given the low number of *Cryptosporidium*‐positive cases (*n* = 2), the observed prevalence of 3.6% is associated with a wide confidence interval and therefore represents an imprecise estimate of the true burden of infection in patients with hematologic malignancies in South Khorasan. As such, our prevalence data should be interpreted with caution and viewed as preliminary rather than definitive. The reliance on a hospital‐based convenience sample also restricts the external validity of the findings, and the results may not be generalizable to all patients with hematologic malignancies in the region or to other settings.

Another methodological limitation is that the nested PCR assay targeted the gp60 gene using primers primarily designed for *C. parvum* and *C. hominis*. While these primers may also amplify gp60 from some other *Cryptosporidium* species, they are not universally sensitive across all described species and genotypes. Therefore, we cannot exclude the possibility that infections with non‐*parvum*/*hominis* species occurred but were not detected by our assay.

## Funding

No funding was received for this manuscript.

## Conflicts of Interest

The authors declare no conflicts of interest.

## Data Availability

Data sharing is not applicable to this article as no datasets were generated or analyzed during the current study.
